# A novel Liesegang-patterned mineralized hydrogel drives bone regeneration with microstructure control

**DOI:** 10.1016/j.mtbio.2025.101775

**Published:** 2025-04-18

**Authors:** Yun Wang, Chao Fang, Li-Bo Mao, Yan-Hui-Zhi Feng, Yu-Feng Meng, Hai-Cheng Wang, Shu-Hong Yu, Zuo-Lin Wang

**Affiliations:** a Department of Oral Implantology and Department of Oral and Maxillofacial Surgery, Stomatological Hospital and Dental School of Tongji University, Shanghai Engineering Research Center of Tooth Restoration and Regeneration, Shanghai, 200072, China; b New Cornerstone Science Laboratory, Department of Chemistry, Institute of Biomimetic Materials & Chemistry, Anhui Engineering Laboratory of Biomimetic Materials, Division of Nanomaterials & Chemistry, Hefei National Research Center for Physical Sciences at the Microscale, University of Science and Technology of China, Hefei, 230026, China; c Institute of Innovative Materials, Department of Chemistry, Department of Materials Science and Engineering, Southern University of Science and Technology, Shenzhen, 518055, China; d Department of Orthopedics, The First Affiliated Hospital of USTC, Division of Life Sciences and Medicine, University of Science and Technology of China, Hefei, 230001, China; e Department of Plant and Environmental Sciences, Weizmann Institute of Science, Rehovot, 7610001, Israel

**Keywords:** Liesegang pattern, Biomimetic structure, Biomimetic mineralization, Alginate, Mineralized hydrogel, Bone regeneration

## Abstract

Bone regeneration remains a critical challenge in modern medicine. Recent advancements have focused on incorporating hierarchical microstructures into biomaterials to enhance osteogenesis. Mineralized hydrogels, while promising, face limitations in precise microstructure control due to technical complexities. In this study, we present a biomimetic hierarchical structural mineralized hydrogel featuring a Liesegang pattern. *In vitro* experiments confirm that it significantly promotes the migration and osteogenic differentiation of bone mesenchymal stem cells (BMSCs). *In vivo* experiments further demonstrate its ability to significantly promote bone regeneration, with newly formed bone closely replicating the hydrogel's architecture. Notably, this hydrogel synthesis strategy eliminates time-consuming fabrication and extensive post-processing, offering a scalable and efficient route for advanced bone-regenerative materials.

## Highlights

• Development of mineralized hydrogels with tailored structures and robust mechanical integrity.
• Use of simple, one-step fabrication technique to create hierarchical architectures that mimic natural bone.
• Comprehensive *in vitro* and *in vivo* evaluations of biocompatibility and osteogenic potential.
• The hierarchical structural hydrogels uniquely templated regenerated bone microstructure.

## Graphical abstract

**Figure:**
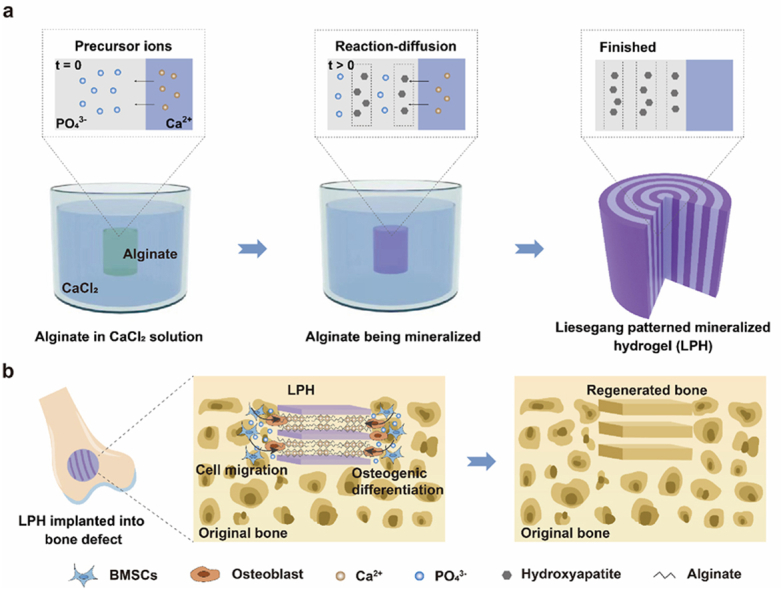

## 1 Introduction

Critical size bone defects resulting from trauma, infection, congenital diseases, or tumors cannot self-heal without clinical intervention [[1], [2], [3]]. Similarly, craniofacial bone loss caused by periodontitis or tooth extraction necessitates bone augmentation to support dental implantation [4,5]. While autografts and allografts face limitations such as donor scarcity and immunogenicity, artificial bone substitutes have emerged as promising alternatives [[6], [7], [8]]. Current research emphasizes the importance of mimicking the natural composition and structure of bone mineral for enhancing bone regeneration [9,10]. However, developing scaffolds that replicate the anisotropic and hierarchical architecture of natural bone remains a formidable challenge. Recent researches indicate that hierarchical structural materials have the potential to provide a distinctive microenvironment that stimulate osteogenesis [[11], [12], [13], [14], [15], [16], [17]], underscoring the critical role of structural design in optimizing bone regeneration outcomes.

Mineralized hydrogel scaffolds have emerged as promising candidates for bone regeneration [18]. Various fabrication techniques have been explored, i.e., incorporation of preformed mineralized particles [19,20], incubation in physiological mineralization solutions [[21], [22], [23]], or biomacromolecules-triggered mineralization [[24], [25], [26]]. However, these methods often result in randomly distributed minerals, lacking the organized microstructure necessary for effective bone regeneration [27,28]. Recently, a nacre-like mineralized hydrogel was developed via the mineralization of a laminated hydrogel matrix [29]. The 3D-printed matrix featured alternating layers with or without alkaline phosphatase, enabling localized calcium phosphate precipitation in enzyme-containing layers, while preventing mineralization in the enzyme-free layers. Despite notable improvements, the multistep fabrication process remains time-consuming, posing a barrier to practical implementation [30].

To overcome the above limitations, a self-assembly-based strategy that simultaneous achieves hydrogel structuralization and mineralization is highly desirable. The Liesegang phenomenon, characterized by the formation of periodic precipitates patterns in diffusion-governed reactions, offers a promising solution [[31], [32], [33], [34], [35]]. Unlike conventional methods, this approach enables rapid top-down structuralization concurrent with mineralization, significantly reducing fabrication time. Moreover, the self-organized periodic patterns address the issue of random mineral distribution observed in previous hydrogel systems, offering a more controlled and biomimetic microstructure.

Inspired by this phenomenon, we present a facile method to fabricate structurally mineralized hydrogel with Liesegang pattern. In this system, self-organized calcium phosphate rings form within an alginate matrix through the reverse diffusion of calcium and phosphate ions. By modulating ion concentrations and pH, both the microstructure and mineral content can be finely tuned, allowing for tailored mechanical and physiological properties. Notably, unlike structural mineralized hydrogels fabricated through multistep methods, this approach eliminates interfacial defects, ensuring superior mechanical integrity and preventing material collapse. *In vivo* experiments reveal that the Liesegang-patterned mineralized hydrogel (LPH) can promote more new bone growth compared to control group. The regenerated bone closely recapitulating the scaffold's architecture. This strategy offers a novel, cell- and therapeutic agent-free approach to designing structural bone-regenerative materials for well-organized bone repair.

## 2 Results

### 2.1 Design and preparation

**Fig. 1:**
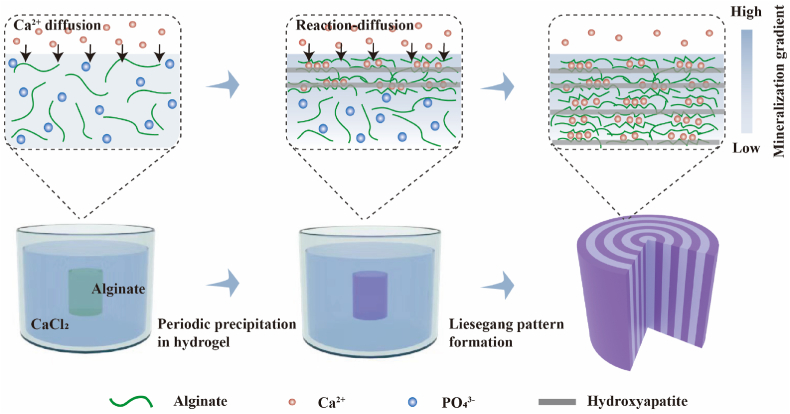
Design and preparation of Liesegang-patterned mineralized hydrogel (LPH). A schematic diagram of the LPH prepared through the non-equilibrium reaction-diffusion process.

The hierarchical structured mineralized hydrogel, designated as LPH, was synthesized through a one-step mineralization process, as illustrated in Fig. 1. Phosphate and calcium ions were selected as the complementary electrolytes, based on their critical role in forming hydroxyapatite-the primary inorganic component of natural bone [2,36,37]. Alginate was chosen as the matrix material due to its demonstrated potential in bone regeneration [[38], [39], [40]]. Previous studies have shown that BMSCs near bone defects migrate towards alginate hydrogels, where they differentiate into osteogenic lineages and initiate the mineralization of the alginate matrix [41,42]. Furthermore, alginate possesses tunable mechanical properties, which are essential for promoting bone regeneration [[42], [43], [44]]. In this process, rather than initially crosslinking the alginate solution with calcium ions to form a hydrogel, phosphate ions were first mixed with the alginate solution as the internal electrolyte. This mixture remained stable until it was immersed in a calcium dichloride solution. Upon immersion, a solidified interfacial layer rapidly formed due to the crosslinking and gelation of alginate by external calcium ions, encapsulating the unsolidified alginate solution inside. This gelatinized coating acted as a container, enabling the diffusion of phosphate and calcium ions and their subsequent precipitation reaction within the encapsulated alginate solution. Since mass transfer in liquid solutions occurs significantly faster than in gels, this strategy greatly accelerated the production of the LPH. Unlike gel-based reaction media, which typically require nearly a week to complete [45,46], this reaction-diffusion process can be accomplished within a single day.

**Fig. 2:**
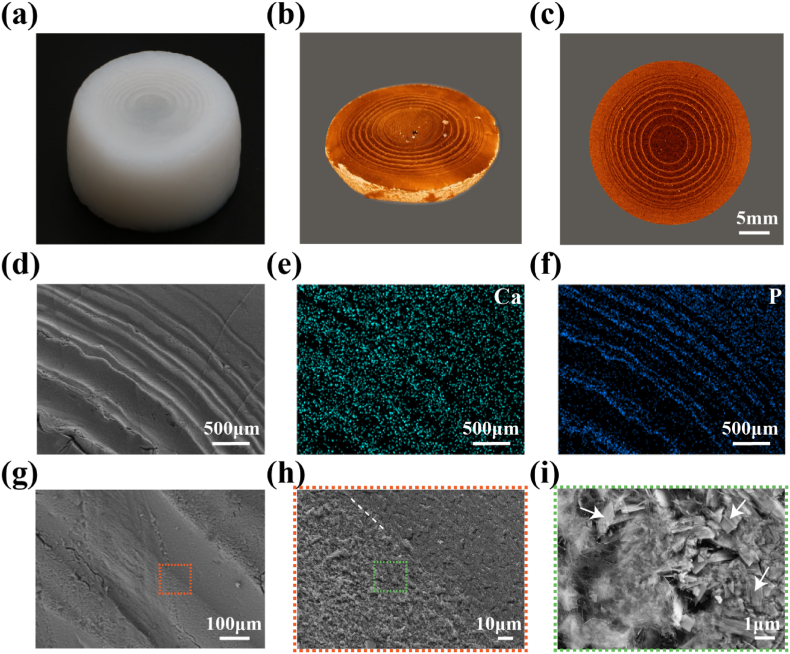
Basic characterizations of the LPH. (a) General observation. (b) Reconstructed three-dimensional Micro-CT image. (c) Cross-section Micro-CT image. (d–f) SEM image and EDX mapping of Ca and P. (g–i) SEM image with different observation magnification. The left of the white dashed line corresponded to highly mineralized area, while the right contained poorly mineralized area. The white arrows refer to the dispersive hydroxyapatite in the polymer network.

As the alginate solution gradually underwent gelation, simultaneous mineralization occurred, ultimately forming a fully solidified white hydrogel (Fig. 2a). The cross-section of the hydrogel exhibited an osteon-like periodic pattern, closely resembling the structure of natural bone. Notably, unlike the multilayered mineralized hydrogel produced through the multistep technique, this one-step strategy effectively mimicked the growth process of natural biological tissues. This approach enabled the formation of a seamless LPH with spatiotemporal self-organization properties. In contrast, the multistep technique often resulted in delamination due to the lack of adhesion between the mineral and the hydrogel layers. The LPH, however, demonstrated significantly enhanced mechanical integrity, with no observable cracks between the mineralized rings and the hydrogel rings (Fig. S1). This hierarchical structured design is expected to facilitated bone repair by closely mimicking the natural bone microenvironment, promoting cell migration, differentiation, and mineralization, thereby meeting the critical requirements for bone regeneration materials.

### 2.2 Hierarchical microstructure and its tunability

To better visualize the internal structure of the LPH, we conducted micro-computed tomography (micro-CT) scanning. The 3D reconstructed images revealed a hierarchical, periodic pattern of alternating bright and dark regions within the hydrogel, corresponding to highly mineralized and poorly mineralized alginate, respectively (Fig. 2b and c; and Movie S1). This pattern, characterized by increasing spacing between the adjacent mineralized rings increased toward the center (Fig. S2), closely follows the spacing law of Liesegang pattern [35,47]. Based on these observations, the hydrogel could be divided in three distinct regions – the continuously mineralized outer area, the middle region with mineralized rings, and the poorly mineralized area in the center. This osteon-like microstructure, resembling the natural architecture of bone, was further supported by scanning electron microscope (SEM) observations (Fig. 2d–g). Energy-dispersive X-ray spectroscopy (EDX) and X-ray diffraction (XRD) analysis confirmed the presence of a calcium phosphate mineral phase, specifically hydroxyapatite, within the mineralized hydrogels (Fig. 2e and f; and Figs. S3 and S4). In addition, Fourier transform infrared spectroscopy (FTIR) further validated the mineral composition, with the LPH exhibiting characteristic phosphate (565, 601 and 1035 cm^−1^) and hydroxyl (3500 cm^−1^) bands (Fig. S5). Furthermore, magnified SEM images showed that the mineralized rings were composed of mineral nanocrystals dispersed in the polymer matrix (Fig. 2i). Importantly, the polymer matrix remained seamless throughout the LPH, with no abrupt compositional changes between the mineralized rings and the adjacent poorly mineralized hydrogel regions (Fig. 2h). This unique structural continuity not only mimics the natural bone microenvironment but also provides the mechanical integrity necessary for bone scaffold applications.

**Fig. 3:**
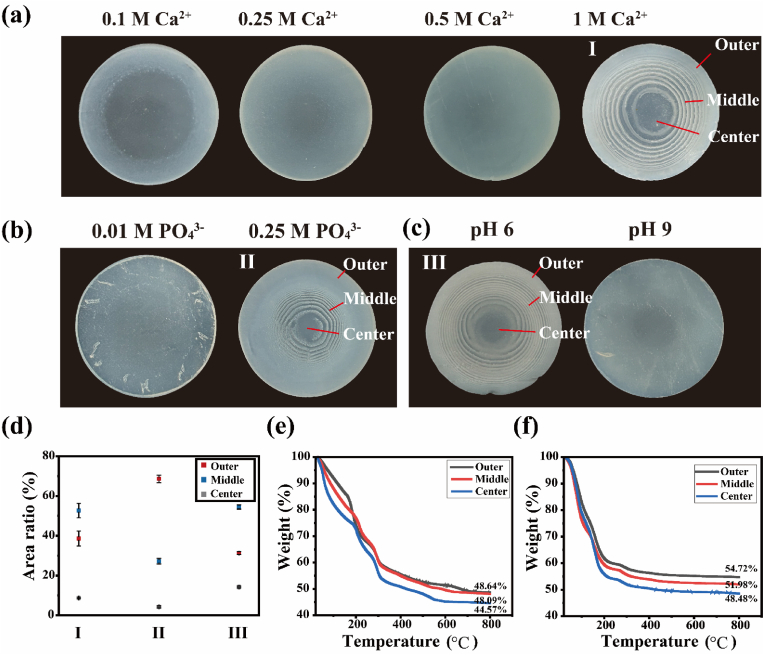
Comprehension of parameters influential in the LPH. (a) Images of mineralized hydrogel complexes prepared at varying concentrations of Ca^2+^ (with other reaction conditions maintained at pH 8, 0.1 M PO_4_^3−^, and room temperature). (b) Images of mineralized hydrogel complexes prepared at varying concentrations of PO_4_^3−^ (with other reaction conditions maintained at pH 8, 1 M Ca^2+^, and room temperature). (c) Images of mineralized hydrogel complexes prepared at varying pH (with other reaction conditions maintained at 0.1 M PO_4_^3−^, 1 M Ca^2+^, and room temperature). (d) Comparative analysis of the area ratios of specific regions across sample I, II and III. (e) Thermogravimetric analysis (TGA) curve of dry sample I. (f) TGA curve of day sample II.

The tunability of the LPH's hierarchical structure was demonstrated through systematic variations in ion concentration, pH, temperature, and polymer matrix composition (Table S1). In a typical sample (sample I), the volume fractions of each area were about 35% (outer), 60% (middle) and 5% (center) respectively (Fig. 3a–d). Both the wet and the dried samples exhibited a gradient of decreasing mineral content from the periphery of sample to its center, a feature critical for mimicking the natural mineral distribution in bone (Fig. 3e and f; and Fig. S6). Adjusting the calcium ion concentration revealed that the periodic pattern formation required an external electrolyte concentration at least one order of magnitude higher than the internal concentration, consistent with classical reaction-diffusion systems (Fig. 3a) [35,48]. Similarly, the concentration of internal anionic electrolytes played a critical role in controlling LPH formation. For example, increasing the phosphate ions concentration shifted the mineralized rings toward the center (Fig. 3b, right sample II), with the volume fractions of approximately 65% (outer), 30% (middle) and 5% (center) respectively (Fig. 3d). This adjustment also increased the overall mineral content by nearly 5% (Fig. 3e and f), highlighting the precise control over mineralization density achievable with this system. However, the insufficient phosphate ion concentration disrupted the reaction-diffusion balance, preventing LPH formation (Fig. 3b, left). In addition, the formation of the LPH proved to be responsive to the pH and temperature. Notably, the spacing of the mineralized rings varied between weakly base (sample I) and weakly acidic (sample III) conditions (Fig. 3a (right), c (left); and Fig. S7), but the LPH failed to form at either neutral pH or highly alkaline pH (pH 9) (Fig. 3c (right); and Fig. S8). Similarly, low temperatures (4 °C) inhibited the LPH formation, as the reduced mineral formation rate also disrupted the balance of reaction and diffusion (Fig. S9). These findings underscore the importance of maintaining a delicate balance between reaction and diffusion dynamics for successful LPH synthesis. Furthermore, the stability of the polymer matrix was essential for LPH formation. Reducing the alginate concentration damaged the internal structure and disrupted the reaction-diffusion system, preventing LPH formation (Fig. S10). This highlights the critical role of the polymer matrix in maintaining structural integrity during mineralization.

The tunable hierarchical structure of the LPH, achieved through precise control of ion concentration, pH, temperature, and polymer matrix composition, offers significant advantages for bone scaffold applications. By closely mimicking the osteon-like microstructure of natural bone, the LPH provides an optimal environment for cell migration, differentiation, and mineralization. The gradient mineral distribution and seamless integration of mineralized and poorly-mineralized regions enhance mechanical stability and bioactivity, making the LPH a promising candidate for bone repair.

### 2.3 Physicochemical properties

**Fig. 4:**
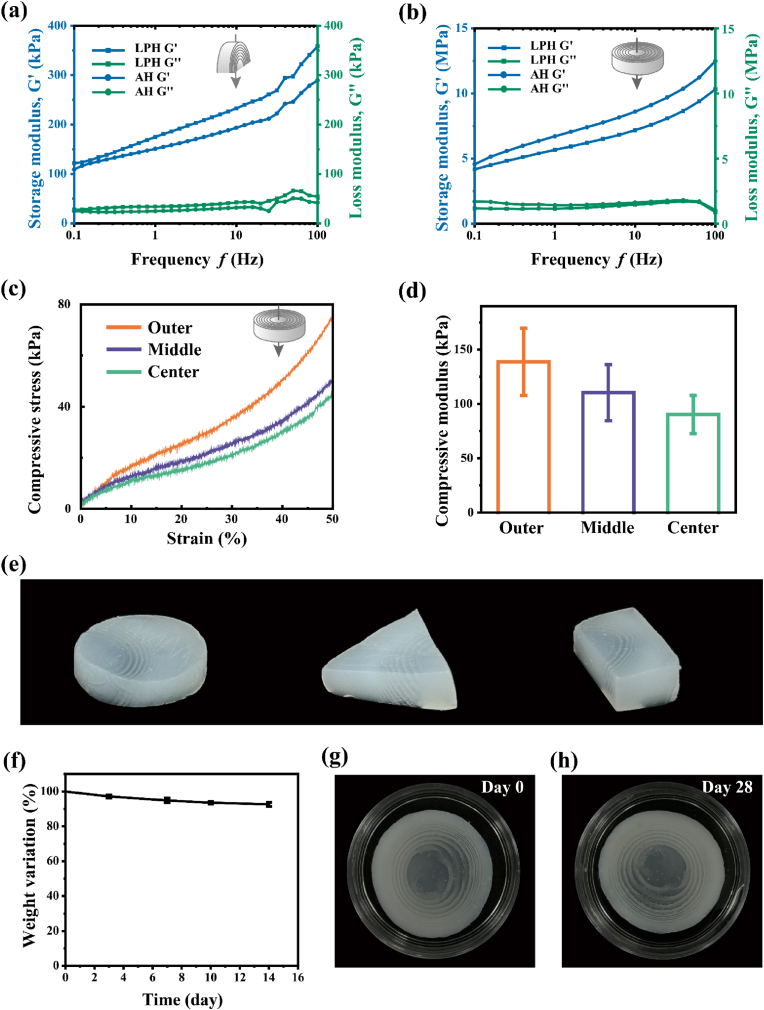
Physical and chemical properties of the LPH. (a, b) Dynamic mechanical properties of the sample I under varying directions of applied force. (c, d) Compressive stress curves and modulus of specific regions within the sample I. (e) The LPH can be divided into arbitrary shapes without delamination. (f) Weight variation of the LPH following a 14-day immersion in deionized water. (g, h) Photographs of the LPH before and after a 4-week immersion in deionized water.

To further understand the properties of the LPH, we test the mechanical properties, providing evidence for the use of hydrogels as scaffolds for tissue engineering. Dynamic mechanical analysis (DMA) was performed on the LPH and alginate hydrogel (AH). The storage modulus (*G′*) is higher than the loss modulus (*G″*), implying the formation of the hydrogels of both LPH and AH (Fig. 4a and b). Furthermore, DMA results revealed that the storage modulus (*G′*) consistently dominated over the loss modulus (*G″*) across the entire tested frequency range (0–100 Hz), demonstrating the hydrogel's robust viscoelastic properties and structural stability under dynamic loading conditions [49]. Moreover, the hydrogel with or without the hydroxyapatite exhibited similar mechanical properties, suggesting that hydroxyapatite did not affect the elastic characteristic or stable polymer network structure of the LPH [15]. This predominance of elastic response (*G′* > *G″*) indicates that the hydrogel network maintains its structural integrity even at high-frequency deformations, a critical characteristic for load-bearing biomedical applications [[50], [51], [52]].

In hydrogel research, compressive properties represent a critical performance metric for practical applications. During operation, hydrogels are routinely subjected to mechanical stresses, including extrusion, friction and other behaviors. Consequently, achieving sufficient mechanical robustness—notably compressive strength—is essential to ensure structural integrity and functional durability *in vivo*. Compared to the AH, the overall compression strength of the LPH remained uncompromised (Fig. S11), indicating the sufficient interfacial adhesive strength to prevent delamination between the hard mineralized hydrogel and the soft poorly mineralized hydrogel regions. This seamless integration is critical for maintaining mechanical integrity under load-bearing conditions, a key requirement for bone scaffold materials. Conspicuously, the compressive modulus of each segment of the LPH increased with mineral content, increasing progressively from the compliant core (90 kPa) to the stiff periphery (140 kPa) (Fig. 4c and d). This mechanical gradient exhibited strong positive correlation with the spatial mineralization profile quantified by micro-CT and TGA analysis, confirming that mineral content directly governs local stiffness variations within the hydrogel architecture. The design of hydrogels with precisely tailored gradient architectures that recapitulate the native tissue's structural and mechanical heterogeneity is critical for their successful clinical translation. Such biomimetic gradient hydrogels can more effectively emulate the transitional zones observed in natural tissue interfaces (e.g., osteochondral tissue with its smooth cartilage-to-bone transition) [53], thereby improving host integration and functional performance in regenerative applications. Additionally, the LPH's robustness allowed it to be easily cut into various shapes without compromising its structure (Fig. 4e), highlighting that they did not disintegrate under the intended conditions of biomedical applications.

Furthermore, the structural stability of the LPH in aqueous environments was evaluated by immersing in deionized water. The results showed that the slowly weight loss within 2 weeks due to hydrogel hydrolysis (Fig. 4f). Despite a slight loss of mass, the LPH maintained its structure without degradation after soaking for up to four weeks (Fig. 4g and h), confirming its exceptional structural stability in aqueous environments. This property is particularly critical for hydrogels, as many tend to swell or degrade in water, compromising their structural integrity and making long-term storage challenging [54,55]. For biomedical applications, maintaining stability in water is essential not only for ease of storage but also for ensuring that the material retains its structural and mechanical properties over an expected period of time. Unlike hydrogels that undergo excessive swelling or rapid degradation, the LPH's durability in water ensures it can provide sustained mechanical support throughout the bone healing process, making it highly suitable for tissue engineering applications.

In summary, the LPH's gradient mechanical properties, seamless integration of mineralized and poorly-mineralized regions, and outstanding mechanical and structure stability collectively make it a highly promising candidate for bone scaffold applications. Its heterogeneous composition and gradient mechanical performance closely mimic the natural structure of bone, creating an optimal environment for cell adhesion, proliferation, and differentiation [53,56,57]. These features, combined with the material's tunability and versatility, underscore its potential to significantly advance bone tissue engineering.

### 2.4 *In vitro* cellular responses of hydrogels

**Fig. 5:**
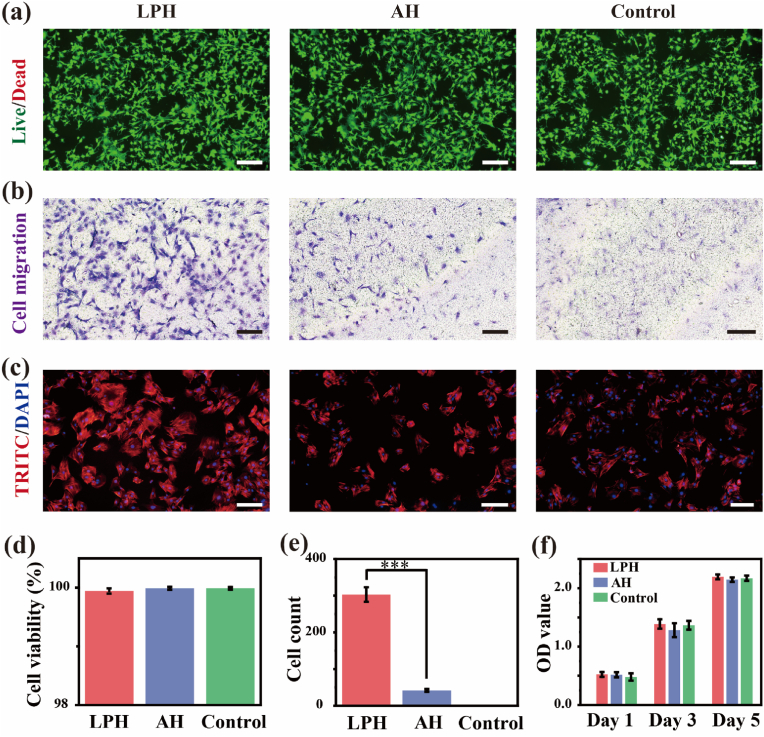
Evaluation of BMSCs biological behaviors co-cultured with the extraction of the LPH and the AH. (a) Live (green)/dead (red) cell staining of BMSCs. Scale bar: 200 μm. (b) Crystal violet staining of the migrated BMSCs. Scale bar: 200 μm. (c) Cytoskeleton and nucleus staining of BMSCs. Scale bar: 200 μm. (d) Cell viability statistics of (a). (e) Cell counts statistics of (b). (f) CCK-8 analysis of BMSCs. Data are presented with mean ± SD (n = 3), ∗*p* ≤ 0.05, ∗∗*p* ≤ 0.01, ∗∗∗*p* ≤ 0.001. (For interpretation of the references to colour in this figure legend, the reader is referred to the Web version of this article.)

The primary requirement for the biomedical application of hydrogels is their biosafety. The biosafety was assessed *in vitro* using rat BMSCs. To evaluate the biosafety of the hydrogels, live (green)/dead (red) cell staining was performed. Optical microscope images revealed minimal dead cells and a live cell proportion exceeding 99% in all groups (Fig. 5a, d), confirming the excellent biocompatibility of the hydrogels. Moreover, a Transwell assay demonstrated a significantly higher migration rate of BMSCs in the LPH group compared to the AH group (Fig. 5b, e), while few cell penetration was observed in the pure medium group. These results highlight the LPH's enhanced pro-migration ability, which is critical for attracting cells to the site of bone repair. The cell spreading behavior of BMSCs was further investigated using nuclei (DAPI) and cytoskeleton (rhodamine phalloidin) staining. After one day of co-culture, BMSCs in the LPH group exhibited better-spread morphology and more elongated, compact actin filaments compared to those in the AH group (Fig. 5c), indicating the LPH's superior affinity for cell adhesion and spreading. Additionally, cell counts performed at 1, 3, 5 days post-co-cultured showed that both sample groups increased gradually over time (Fig. 5f), further confirming the biocompatibility of the hydrogels.

**Fig. 6:**
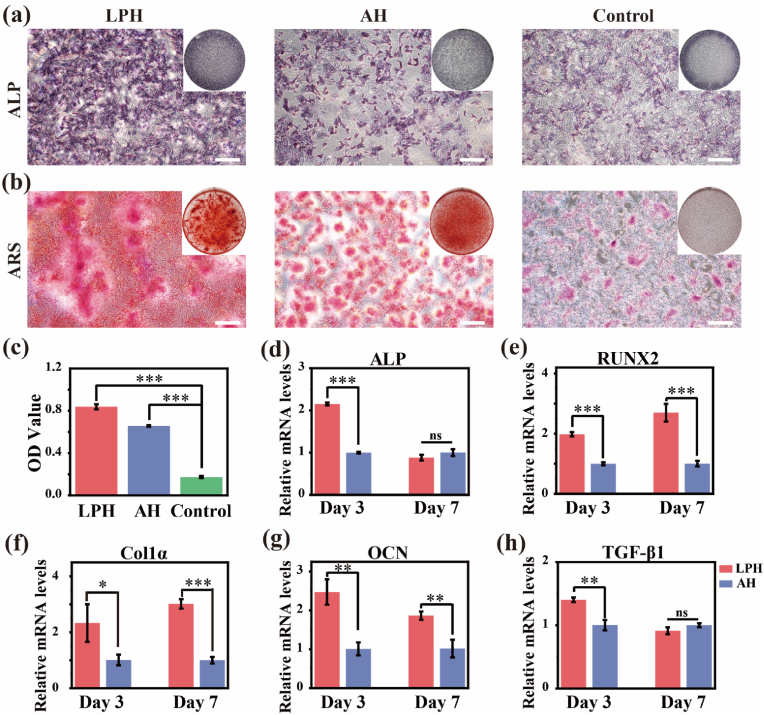
Evaluation of BMSCs osteogenic ability co-cultured with the extraction of the LPH and the AH. (a) ALP staining of BMSCs after 7 days of culture. Scale bar: 500 μm. (b) ARS of BMSCs after 21 days of culture. Scale bar: 500 μm. (c) Quantification analysis of ARS results of (b). (d–h) Analysis of relative mRNA expression level of BMSCs. Data are presented with mean ± SD (n = 3), ∗*p* ≤ 0.05, ∗∗*p* ≤ 0.01, ∗∗∗*p* ≤ 0.001.

To assess the osteogenic-facilitated potential of the hydrogels, the osteogenic performances of the BMSCs were analyzed for alkaline phosphatase (ALP) activity, extracellular matrix (ECM) mineralization, and osteogenic-related genes expression level. ALP staining, which reflects early osteogenic differentiation, demonstrated the higher ALP activity of the LPH group compared to the AH group (Fig. 6a). Alizarin red staining (ARS) and semiquantitative analysis demonstrated greater ECM mineralization in the LPH group, as evidenced by the higher density of calcified nodules (Fig. 6b and c). These findings suggest that the LPH significantly enhances the mineralization capacity of BMSCs, a key step in bone formation. Furthermore, the gene expression analysis of ALP, RUNX2, Col1*α*, OCN, and TGF-*β*1 were performed to validate the LPH's osteogenic-facilitated potential. After 3 days and 7 days of co-culture, BMSCs in the LPH group exhibited higher expression levels of most osteogenic genes compared to those in the AH group (Fig. 6d–h). In summary, the LPH's excellent biocompatibility, enhanced cell migration and adhesion, and strong osteogenic-promoting properties make it a highly promising material for bone defect repair. By creating a favorable microenvironment for BMSCs to proliferate, differentiate, and mineralize, the LPH demonstrates significant potential to advance bone tissue engineering.

### 2.5 *In vivo* evaluation of LPH for bone regeneration using a rat femoral defect model

**Fig. 7:**
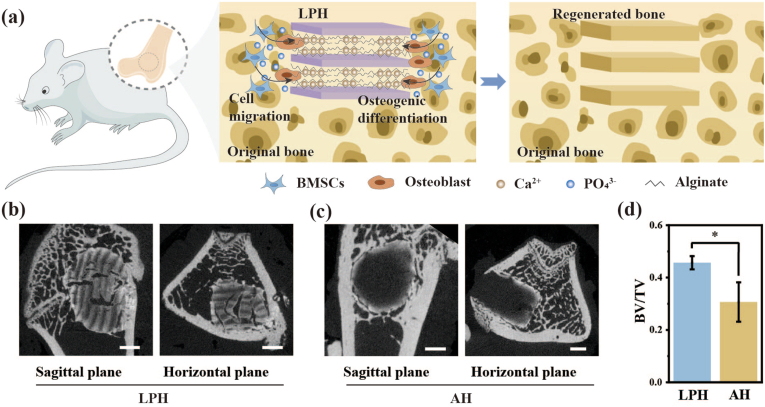
Evaluation of the osteogenic ability of LPH on bone regeneration. (a) A schematic diagram of the construction of the femur defect model in Sprague-Dawley (SD) rats and its underlying osteogenic process. (b, c) Micro-CT images of the femoral defects in SD rats following a two-month implantation of the LPH and the AH. Scale bar: 1 mm. (d) Quantitative analysis of new bone volume fraction based on micro-CT data. Data are presented with mean ± SD (n = 3), ∗*p* ≤ 0.05, ∗∗*p* ≤ 0.01, ∗∗∗*p* ≤ 0.001.

**Fig. 8:**
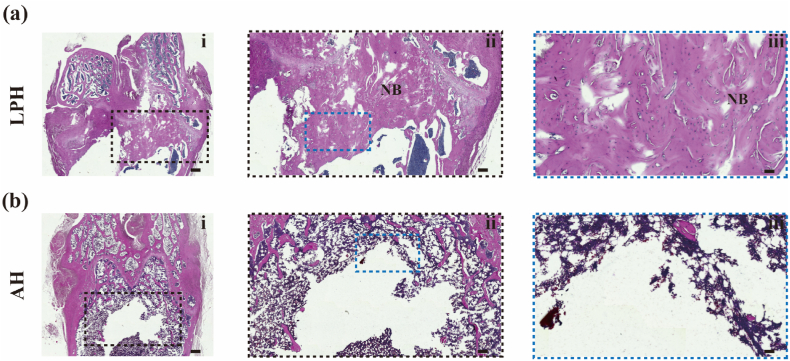
Histological evaluation of decalcified sections stained with H&E (the black dotted rectangles represent bone defects areas). (a) LPH group; (b) AH group (Figure ii is the enlarged image of the black dotted rectangles in Figure i; Figure iii is the enlarged image of the blue dotted rectangles in Figure ii; NB means new bone.). Scale bar: (i) 500 μm; (ii) 200 μm; (iii) 50 μm. (For interpretation of the references to colour in this figure legend, the reader is referred to the Web version of this article.)

To validate the *in vivo* osteogenic potential of materials, an 8-week Sprague Dawley (SD) rat femur defect model was employed (Fig. 7a). Micro-CT images showed the presence of a Liesegang-patterned structure in the regenerated bone area (Fig. 7b), demonstrating an extraordinary phenomenon that the microstructure of the original LPH was almost “transcribed” into the newly formed bone. Importantly, the mineralization degree of artificial mineralized hydrogels, such as the LPH, is inherently lower than that of natural bone. As a result, the contrast of the hydrogel in micro-CT imaging is significantly weaker than that of bone. The observation of regions with bone-like contrast in the micro-CT images thus confirms the presence of newly formed bone rather than the original hydrogel. This suggests that the hierarchical design of the LPH not only supports bone regeneration but also guides the microstructure of the regenerated tissue. In contrast, defects treated with the AH showed poorly regeneration, primarily restricted to the periphery of the implanted material (Fig. 7c). Quantitative analysis of the micro-CT data, specifically the bone volume/tissue volume (BV/TV) ratio, confirmed that the LPH group exhibited significantly enhanced new bone formation compared to the AH group (Fig. 7d). Histological examination of the decalcified bone sections using hematoxylin and eosin (H&E) staining further corroborated the micro-CT findings. The results demonstrated a significantly higher quantity of newly formed bone in the LPH group compared to the AH group, consistent with the micro-CT observations (Fig. 8). Dense and mature woven bone was evident in the LPH group; however, due to the decalcification process, the histological staining did not fully capture the Liesegang patterns observed in the micro-CT images. This discrepancy highlights the complementary nature of micro-CT and histological analyses in evaluating bone regeneration. Those findings underscore the critical role of the LPH's specific periodic structure in promoting bone mass augmentation and microstructural organization during regeneration. The ability of the LPH to "transcribe" its structural design into the regenerated bone demonstrates the potential of tuning the architecture and properties of newly formed bone through the structural design of bone repair materials. By leveraging mineralized hydrogels with hierarchical structures, it is possible to create materials that not only enhance osteogenesis but also reproduce the natural microstructure of bone, offering a promising strategy for advanced bone tissue engineering.

## 3 Conclusion

Increasing attention is being directed towards incorporating biomimetic structures into bone repair materials to enhance bone regeneration [58,59]. Innovative biomimetic structural materials, which employ cell-free or therapeutic agent-free strategies to promote bone regeneration, have emerged as a promising paradigm in the field. Among these, mineralized hydrogels stand out as a highly promising material for bone regeneration, as their biomimetic structural design can significantly enhance their ability to promote bone repair [18]. Currently, mineralized hydrogels are primarily synthesized via three approaches, i.e., incorporation of preformed mineral particles, immersion in mineral solutions, and biomacromolecule-induced mineralization [60]. However, achieving internal structuring in mineralized hydrogels remains a significant challenge, particularly in replicating the complex hierarchical architecture of natural bone. To address this challenge, our study developed a straightforward and efficient mineralization strategy based on the non-equilibrium reaction-diffusion theory. This method successfully emulates the formation process of the concentric lamellar structure of osteon under mild physiological conditions. By establishing opposing ion concentration gradients inside and outside the cylindrical precursor solution, we achieved a concentric ring-shaped spatiotemporal self-organization pattern of calcium phosphate. This approach does not require complex instrumentation, highlighting its operational simplicity and cost-effectiveness, which are significant practical advantages for scalable applications.

Notably, the current development of bone repair materials predominantly focuses on bone mass augmentation [[61], [62], [63]]. While commonly used calcium phosphate-based powdered grafts can promote bone regeneration, the resulting bone often exhibits a granular morphology and lacks the structural characteristics of natural bone [64]. This is a critical limitation, as bone is a hierarchically structured biological material, and its microstructure is essential for its mechanical and physiological functions [65,66]. Therefore, the microstructure of the regenerated bone should be carefully designed to mimic natural bone. Fortunately, mineralized hydrogel scaffolds with specific architectures have shown promise in regulating the intrinsic structure of regenerated bone [18]. The hierarchically structured mineralized hydrogel we have designed is anticipated to provide an effective solution to this challenge, offering a material that not only promotes bone regeneration but also guides the formation of a biomimetic microstructure.

In summary, leveraging a non-equilibrium reaction-diffusion process, we report the facile one-step preparation of the LPH, which exhibits a well-organized hierarchical concentric microstructure. The LPH integrates several key advantages, including the excellent mechanical integrity, structure stability, tunable microstructure and composition, biocompatibility, and osteogenic-facilitated capability. Importantly, the LPH can precisely template the microstructure of the regenerated bone, bridging the gap between synthetic materials and natural bone architecture. This strategy paves the way for the development of hierarchical structurally mineralized hydrogels with significant potential for bone repair applications.

## 4 Experimental section

### 4.1 Materials

All chemicals were utilized without further purification. Alginate, Na_2_HPO_4_, and CaCl_2_·2H_2_O were purchased from Aladdin (Shanghai, China). NaOH, HCl, and acetic acid were purchased from National Pharmaceuticals (Shanghai, China). Cell culture medium (α-MEM) and penicillin-streptomycin solution were sourced from Hyclone (Logan, UT, USA). Fetal bovine serum was acquired from Sangon (Shanghai, China). Trypsin was purchased from Gibco (Grand Island, NY, USA). PBS solution and 4% paraformaldehyde solution were supplied by Labgic Technology (Beijing, China). TRITC phalloidin was obtained from Solarbio (Beijing, China). Cell Counting Kit-8 (CCK-8), Calcein/PI Cell Viability and Cytotoxicity Assay Kit, Crystal Violet Staining Solution, BCIP/NBT Alkaline Phosphatase Color Development Kit and Alizarin Red S (ARS) Staining Kit for Osteogenesis were acquired from Beyotime (Shanghai, China). FastPure Cell/Tissue Total RNA Isolation Kit V2 was purchased from Vazyme (Nanjing, China). Quantitative Real-time PCR (RT-qPCR) Primers were sourced from Tsingke (Beijing, China). The reverse transcription kit and Hieff qPCR SYBR Green Master Mix were obtained from were purchased from Yeasen (Shanghai, China).

### 4.2 Preparation of the Liesegang patterned mineralized hydrogel (LPH)

Mineralized hydrogels were prepared following the formulation detailed in Table S1. Na_2_HPO_4_ was dissolved in deionized water to create a homogeneous solution, with pH adjustment performed using HCl. Subsequently, alginate was incorporated into the previous solution and stirred until a homogeneous solution was achieved. CaCl_2_·2H_2_O was also dissolved in deionized water to prepare a uniform CaCl_2_ solution. 25 mL of alginate solution was poured into a glass mold and fully immersed in the CaCl_2_ solution. A thin film rapidly developed on the surface of the solution, encapsulating the inner solution, which subsequently released from the mold. The reaction was allowed to continue in the CaCl_2_ solution until completed gelation occurred.

### 4.3 Preparation of the pure alginate hydrogel (AH) without periodic pattern

The homogeneous 2% alginate solution and 1 M CaCl_2_ solution were prepared. To create the pure alginate hydrogel, we adhered to the previous outlined procedures.

### 4.4 Preparation of multilayer mineralized hydrogel using the multistep technique

A 0.1 M Na_2_HPO_4_ solution was prepared and adjusted to pH 8 with dilute HCl. Subsequently, a 2% alginate was added and stirred until a homogeneous solution was obtained, denoted as solution A. An additional 2% alginate solution, designated as solution B, was also prepared. Furthermore, a 1 M CaCl_2_ solution was prepared. Pure alginate hydrogel core was initially fabricated and subsequently coated layer by layer with both the mineralized hydrogel layer derived from solution A and the pure alginate hydrogel layer originating from solution B.

### 4.5 Characterizations

#### 4.5.1 Structural characterization of the LPH

To facilitate a detailed examination of the internal structure, the LPH was subjected to micro-CT (Xray-Zeiss-Xradia-520-Versa). Image J software was employed for the analysis of micro-CT images, yielding critical insights into the spatial distribution and organization of these structures. Furthermore, to quantitatively assess the area proportions among 3 regions (outer, middle and center) within the LPH, photographs were analyzed using Image J.

To investigate the intricate periodic morphology, the LPH was dried by supercritical drying (Leica EM CPD 300). The Scanning electron microscope (SEM) (Carl Zeiss GeminiSEM 450) images were obtained at an accelerating voltage of 5 kV. Energy-dispersive X-ray spectroscopy (EDX) (OXFORD Ultim Max100) analysis was conducted at an accelerating voltage of 15 kV to determine the elemental composition of the LPH. The composition of the LPH was confirmed by Fourier Transform Infrared Spectroscopy (FTIR) (Thermo Nicolet 6700). The mineral phase in the LPH was characterized by X-ray diffraction (XRD) (Philips X'Pert Pro Super X-ray diffractometer) analysis. In addition, observations of the wet structure of the LPH, especially within the Liesegang ring area, were conducted using a polarizing microscope (OLYMPUS, TH4-200).

#### 4.5.2 Mineral content of the LPH

The mineral content of the LPH was evaluated through thermogravimetric analysis (NETZSCH TG 209 F1 Libra). The LPH sample was divided into 3 sections according to the outer (the continuously mineralized outer area), middle (the mineralized ring area in the middle), and center (the poorly mineralized area in the center) area. Each section underwent thermogravimetric analysis both prior to and following drying. In parallel, the AH sample also underwent thermogravimetric analysis before and after drying as a control. The analyses were conducted under a nitrogen atmosphere to safeguard the samples. The temperature was incrementally increased from room temperature to 800 °C at a rate of 10 °C/min.

#### 4.5.3 Mechanical property test of the LPH

To evaluate the gradient compression strength of the LPH, the samples were categorized into rectangular blocks based on 3 distinct regions, i.e. the outer area (continuously mineralized area), the middle area (mineralized ring area), and the center (poorly mineralized area) for subsequent compression testing (CITEMA 2200). The applied compression force was oriented perpendicular to the concentric circle direction. The compression test was conducted at a strain rate of 1 mm/min, with the termination occurring upon reaching a strain of 50%. Eight samples were tested in each group. The difference in compressive strength is compared to the compressive strength value at a strain of 50%. The compression modulus is calculated by calculating the slope of the stress-strain curve within 5% strain. For comparative analysis between the LPH and the AH regarding compressive performance (TA Q800 DMA), samples were sectioned to an approximately height of 9 mm. Here, the compression force was aligned parallel to the plane of concentric circles at a loading rate of 0.5 N/min, ceasing once a load of 18 N was achieved. Two samples were tested in each group. To investigate differences in deformation under dynamic loading conditions between the LPH and the AH during compression, additional samples measuring approximately 9 mm in height were prepared with similar alignment for force application. The test utilized an amplitude of 20 μm across a frequency range from 0.1 Hz to 100 Hz (TA Q800 DMA). Five samples were tested in each group. Furthermore, dynamic deformation characteristics perpendicular to the concentric circle orientation were also evaluated, these involved samples approximately 2 mm in height subjected to an amplitude of 10 μm within a frequency spectrum ranging from 0.1 Hz to 100 Hz.

#### 4.5.4 Chemical stability of the LPH

To evaluate the chemical stability of the LPH in deionized water, the hydrogel was sectioned into approximately 2 mm slices, and its initial mass was recorded. Subsequently, the sample was immersed in 1 mL of deionized water at ambient temperature. At specified time intervals, each sample were then retrieved from the water and gently remove any excess water on the surface with filter paper. The mass of each sample was recorded at each time point to monitor any variations over time.

### 4.6 Cell experiment

#### 4.6.1 Isolation and culture of male Sprague–Dawley (SD) rat bone marrow mesenchymal stem cells (BMSCs)

BMSCs were isolated from the femur of 3-week male SD rats and collected in the growth medium consisted of α-MEM supplemented with 10% fetal bovine serum and 1% penicillin. Cells were passaged upon reaching approximately 80% confluency. The cells were kept in a humidified incubator at 37 °C with 5% CO_2_, and the culture medium was refreshed every 2–3 days. For subsequent biology experiments, BMSCs from passages 2 to 4 were utilized.

#### 4.6.2 Cell toxicity assay

The proliferation capacity of BMSCs cultured in the conditioning culture medium prepared with hydrogel extracts was assessed using the CCK-8 assay. Specifically, the hydrogel was immersed in the cell culture medium and incubated at 37 °C for 24 h to obtain the extracts. The conditioned culture medium consisted of 50% hydrogel extract mixed with 50% α-MEM, which was utilized for all subsequent cell experiments. BMSCs were plated in a 96-well plate (5 × 10^3^ cells/well, n = 5/group) and cultured in each conditioning medium for durations of 1, 3, and 7 days. The experimental group was co-cultured with a conditioned culture medium, while the control group was cultured with α-MEM alone. Cell viability at each time point was determined using the CCK-8 assay, with quantification performed by measuring the optical density (OD) at 450 nm using a microplate reader (BERTHOLD, Tristar 3).

The cytotoxicity of the hydrogel on BMSCs was evaluated using the Calcein-AM/PI cell viability and cytotoxicity assay. BMSCs were seeded in a 24-well plate (5 × 10^4^ cells/well, n = 3/group) and cultured in various conditioning medium for 3 days. The control group was maintained in α-MEM. The Calcein-AM/PI assay were performed to quantify the proportion of viable versus non-viable cells, with ImageJ software employed for accurate enumeration of live and dead cell populations.

#### 4.6.3 Observation of cell morphology co-cultured with hydrogel extracts

BMSCs were plated in a 24-well plate (5 × 10^4^ cells/well, n = 3/group) and cultured in various conditioning medium for a duration of 24 h. The control group was maintained in α-MEM. Following the incubation period, the cells were fixed with 4% paraformaldehyde (PFA) for 30 min. Subsequently, they were stained with TRITC for 30 min and DAPI for an additional 5 min at 37 °C. After thorough washing with PBS, cellular observations were conducted using a fluorescence microscope (OLYMPUS, TH4-200).

#### 4.6.4 Observation of cell migration ability co-cultured with hydrogel extracts

The migratory capacity of BMSCs was evaluated using 6.5 mm transwell plates equipped with 8.0 μm pore polycarbonate membrane inserts. Following a 24-h starvation period, BMSCs were seeded in the upper chambers of a 24-well plate (2 × 10^4^ cells/well, n = 3/group). The lower chambers contained media from 3 groups, i.e. α-MEM supplemented with 50% LPH extract, αMEM supplemented with 50% AH extract, and plain α-MEM as control. After incubation at 37 °C for an additional 24 h, BMSCs were fixed using 4% paraformaldehyde (PFA). Non-migrated BMSCs were whipped with cotton swabs, while the migrated BMSCs were stained by crystal violet for 20 min, and subsequently counted in 3 random fields under microscope.

#### 4.6.5 ALP staining and alizarin red staining (ARS)

BMSCs were seeded in a 6-well plate (1 × 10^5^ cells/well, n = 3/group) and cultured in various conditioning medium containing osteogenic induction components. The control group was co-cultured with α-MEM containing with the same components. The media were refreshed every 3 days. On day 7, an ALP activity was assessed using an ALP staining kit. On day 21, mineralization was evaluated using ARS staining. For both procedures, the cells were fixed with 4% PFA and subsequently washed with PBS. The incubating solution was applied to the plates for 1 h and rinsed again with PBS. The staining results were observed under a fluorescence microscope. Furthermore, to quantify mineralization, ARS-stained cultures were treated with 10% acetic acid for 30 min at room temperature to release calcium-bound alizarin red. The absorbance of the solution was measured at 450 nm using a microplate reader (BERTHOLD, Tristar 3), followed by statistically analysis.

#### 4.6.6 RT-qPCR analysis

Total RNA was extracted from the cells using a Total RNA Isolation Kit, followed by reverse transcribed with a PrimeScript RT Reagent Kit. The resulting cDNA was then subjected to and RT-qPCR analysis employing SYBR Mix on a LightCycler System. The relative mRNA expression levels were quantified by the 2^−ΔΔCt^ method after normalization to GAPDH. The primer sequence of osteogenesis-relative genes is shown in Table S2.

### 4.7 *In vivo* experiment

#### 4.7.1 SD rat femur defects repair experiment

All animal procedures were conducted in accordance with the guidelines established by the Institutional Animal Care and Use Committee of Tongji University, and received approval from the Ethics Committee of the Affiliated Stomatology Hospital of Tongji University (Approval No. 2024-DW-60, Shanghai, China). Male SD rats aged 8–10 weeks were selected. Following anesthesia, the skin over the knee joint was sterilized and incised to expose the femoral condyle. A 3 mm diameter and 3 mm depth defect was created. Subsequently, the prepared LPH and the AH plugs (diameter 3 mm, length 3 mm) were implanted into the defects. After repositioning the periosteum and muscles, intermittent suturing of the skin was performed, followed by thorough cleaning and sterilization of the wound area. Each experimental group comprised 3 rats. Postoperative antibiotic treatment was administered for a duration of 3 days. Tissue samples were collected after 2 months for analysis, with micro-CT imaging (Scanco Medical, Switzerland) employed to assess new bone formation.

#### 4.7.2 Hematoxylin and eosin (H&E) staining

The decalcified bone tissue was serially sectioned at a thickness of 4 μm. Following graded dehydration, the sections were subjected to hematoxylin and eosin staining. The slides were then cover-slipped and examined under a microscope (Ocus scan).

### 4.8 Statistical analysis

All biology experiments were repeated in triplicate, and the data were presented as the mean ± standard deviation (mean ± SD, n ≥ 3). The graphs were produced using Origin 2021 software. Data from experiments were analyzed with SPSS software (IBM SPSS Statistics, v27.0). Unpaired two-tailed Student's *t*-test is used between two groups, and *P*-values <0.05 considered statistically significant. (∗*p* ≤ 0.05; ∗∗*p* ≤ 0.01; ∗∗∗*p* ≤ 0.001).

## CRediT authorship contribution statement

**Yun Wang:** Writing – original draft, Supervision, Methodology, Investigation, Data curation. **Chao Fang:** Writing – review & editing, Investigation. **Li-Bo Mao:** Writing – review & editing, Project administration, Funding acquisition, Conceptualization. **Yan-Hui-Zhi Feng:** Project administration. **Yu-Feng Meng:** Writing – review & editing, Funding acquisition. **Hai-Cheng Wang:** Project administration. **Shu-Hong Yu:** Writing – review & editing, Supervision, Resources, Funding acquisition, Conceptualization. **Zuo-Lin Wang:** Writing – review & editing, Supervision, Resources, Funding acquisition, Conceptualization.

## Declaration of competing interest

The authors declare the following financial interests/personal relationships which may be considered as potential competing interests: Zuolin Wang reports financial support was provided by the Original Exploration Program of the 10.13039/501100001809National Natural Science Foundation of China. Zuolin Wang reports financial support was provided by the 10.13039/501100012166National Key Research and Development Program of China. Libo Mao reports financial support was provided by the Strategic Priority Research Program of the 10.13039/501100002367Chinese Academy of Sciences. Yufeng Meng reports financial support was provided by the 10.13039/501100001809National Natural Science Foundation of China. Yufeng Meng reports financial support was provided by the Double First-Class University Construction Fund from USTC. Shuhong Yu reports financial support was provided by the Major Basic Research Project of Anhui Province. Shuhong Yu reports financial support was provided by New Cornerstone Science Foundation. If there are other authors, they declare that they have no known competing financial interests or personal relationships that could have appeared to influence the work reported in this paper.

## Data Availability

Data will be made available on request.
