# Supplementary material for: A novel Liesegang-patterned mineralized hydrogel drives bone regeneration with microstructure control
Source: Mater Today Bio. 2025 Apr 18;32:101775. doi: 10.1016/j.mtbio.2025.101775 (PMC12053635; doi:10.1016/j.mtbio.2025.101775)
Supplement: Multimedia component 1 [file mmc1.docx]

Supporting Information

A Novel Liesegang-Patterned Mineralized Hydrogel Drives Bone Regeneration with Microstructure Control

Yun Wang^a,1^, Chao Fang^d,1^, Li-Bo Mao^b^, Yan-Hui-Zhi Feng^a^, Yu-Feng Meng^b,e^, Hai-Cheng Wang^a^, Shu-Hong Yu^b,c^*, Zuo-Lin Wang^a^*

a Department of Oral Implantology and Department of Oral and Maxillofacial Surgery, Stomatological Hospital and Dental School of Tongji University, Shanghai Engineering Research Center of Tooth Restoration and Regeneration, Shanghai 200072, China

b New Cornerstone Science Laboratory, Department of Chemistry, Institute of Biomimetic Materials & Chemistry, Anhui Engineering Laboratory of Biomimetic Materials, Division of Nanomaterials & Chemistry, Hefei National Research Center for Physical Sciences at the Microscale, University of Science and Technology of China, Hefei 230026, China

c Institute of Innovative Materials, Department of Chemistry, Department of Materials Science and Engineering, Southern University of Science and Technology, Shenzhen 518055, China

d Department of Orthopedics, The First Affiliated Hospital of USTC, Division of Life Sciences and Medicine, University of Science and Technology of China, Hefei, 230001, China

e Department of Plant and Environmental Sciences, Weizmann Institute of Science, Rehovot 7610001, Israel

^1^ These authors contributed equally to this work.

* Corresponding authors.

E-mail: zuolin@tongji.edu.cn; shyu@ustc.edu.cn

| 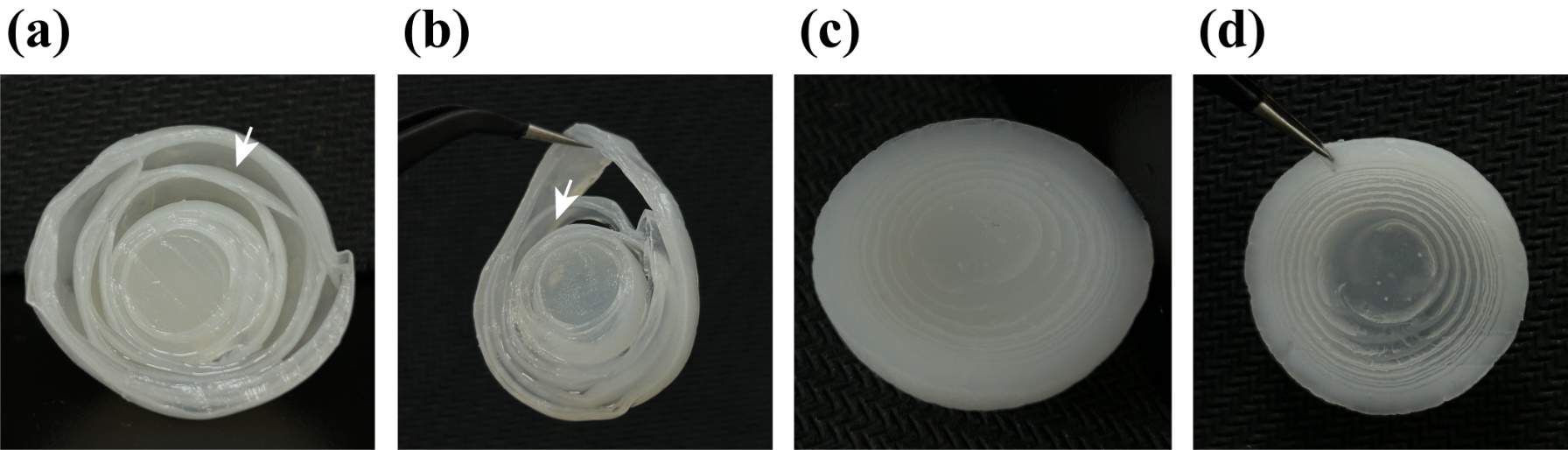 |
| --- |
| **Figure S1** A comparative analysis of multilayer mineralized hydrogel using various techniques. (a, b) Cross-section photograph of the multilayer mineralized hydrogel, obtained through a multistep technique (white arrows indicate interlayer interface debonding). (c, d) Cross-section photographs of the Liesegang-patterned hydrogel (LPH). |

| 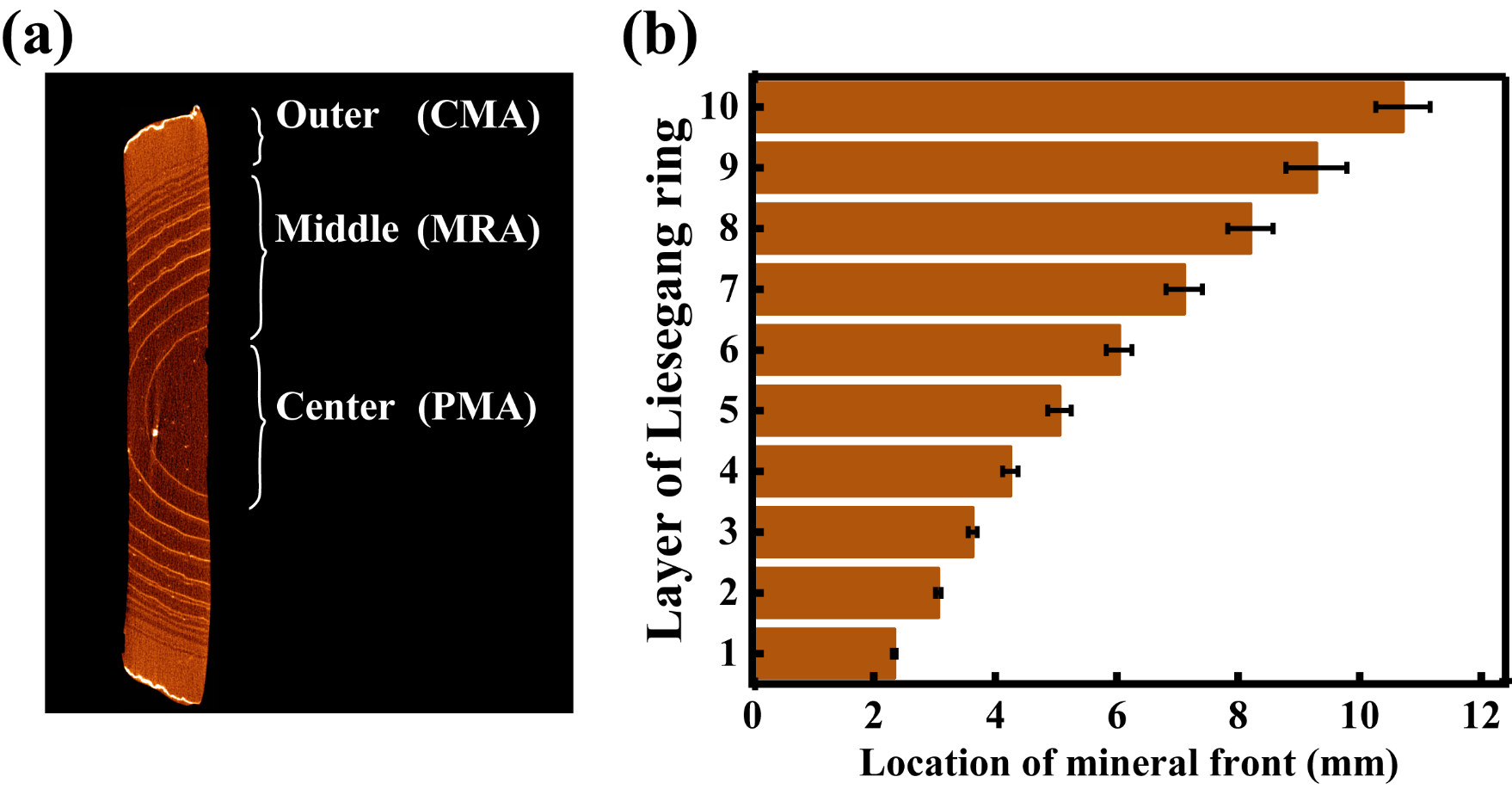 |
| --- |
| **Figure S2** Micro-CT sectional image of the LPH and location of mineral front statistics. (a) Micro-CT sectional image of the LPH. (b) Statistical analysis of mineral front locations within the LPH. CMA refers to outer-continuously mineralized area, MRA refers to middle-mineralized ring aera, and PMA refers to center-poorly mineralized aera. |
|  |

| 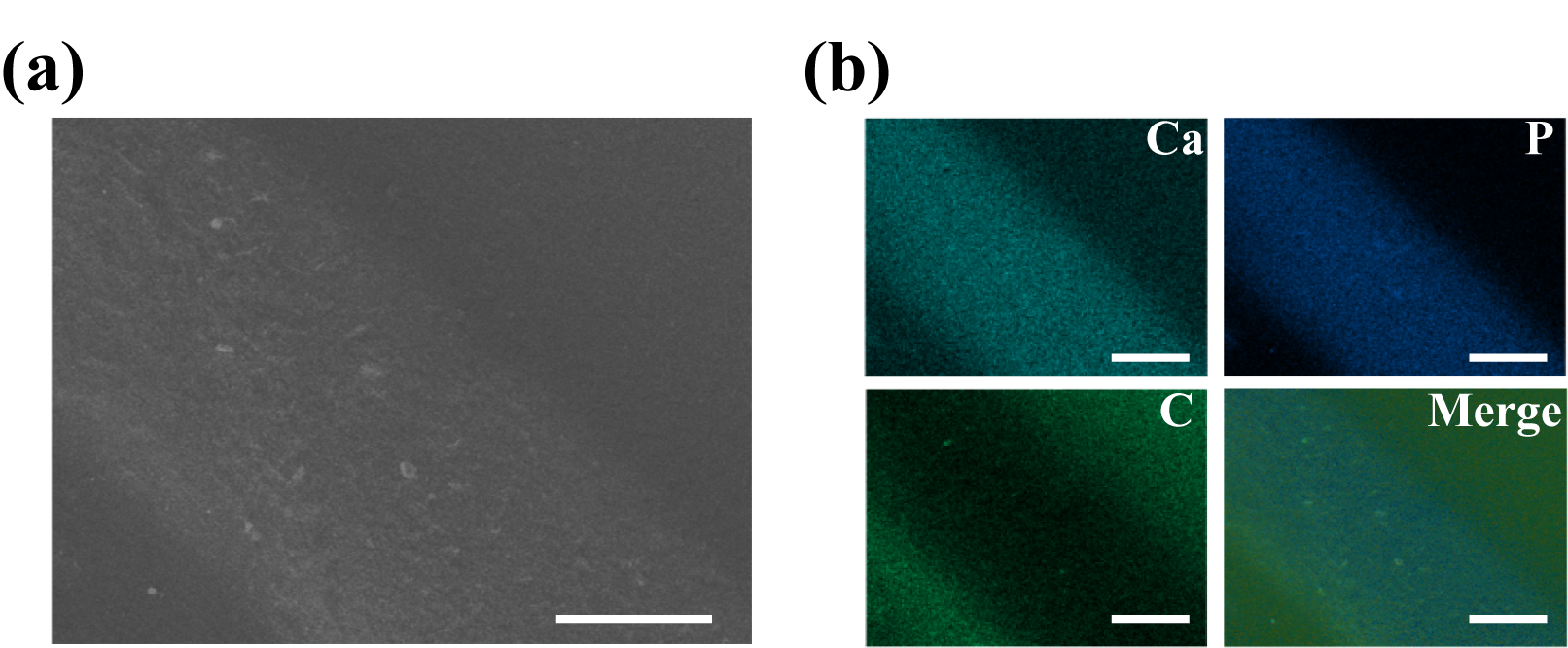 |
| --- |
| **Figure S3** Magnifying SEM image and EDX mapping of the LPH. (a) Magnifying SEM image of the LPH, Scale bar: 50 μm. (b) EDX mapping of (A). Scale bar: 50 μm. |

| 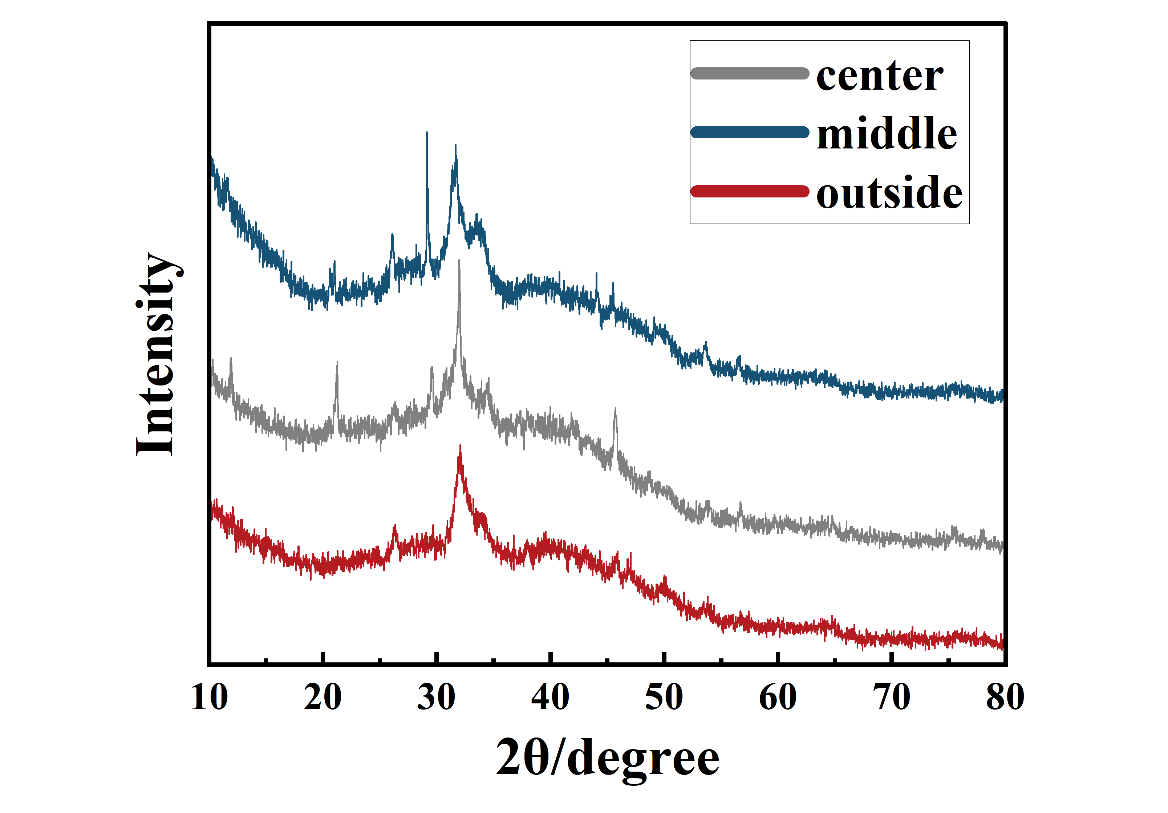 |
| --- |
| **Figure S4** XRD analysis of 3 regions of the LPH. |

| 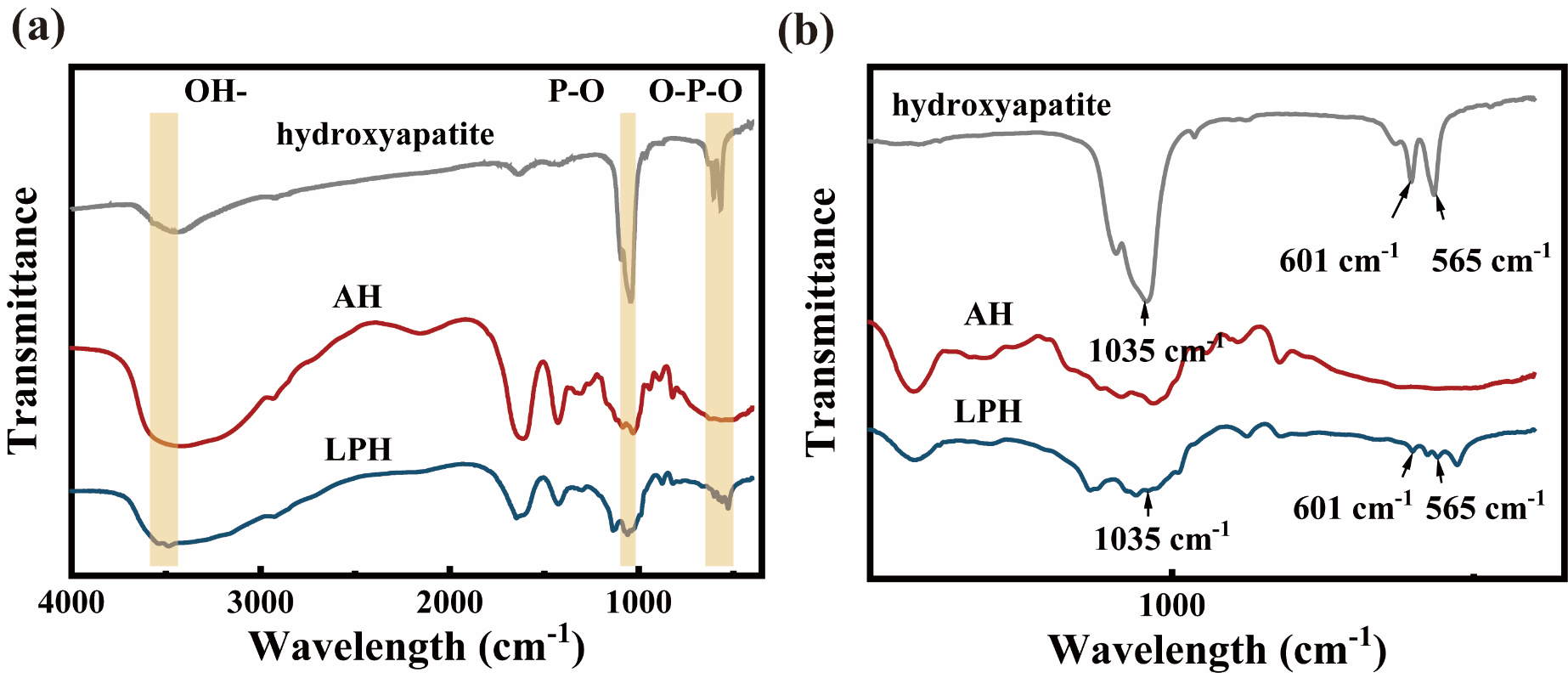 |
| --- |
| **Figure S5** FTIR analysis of the AH and the LPH. (a) FTIR analysis of the AH and the LPH. (b) Magnified of FTIR analysis curve of (a). |

| 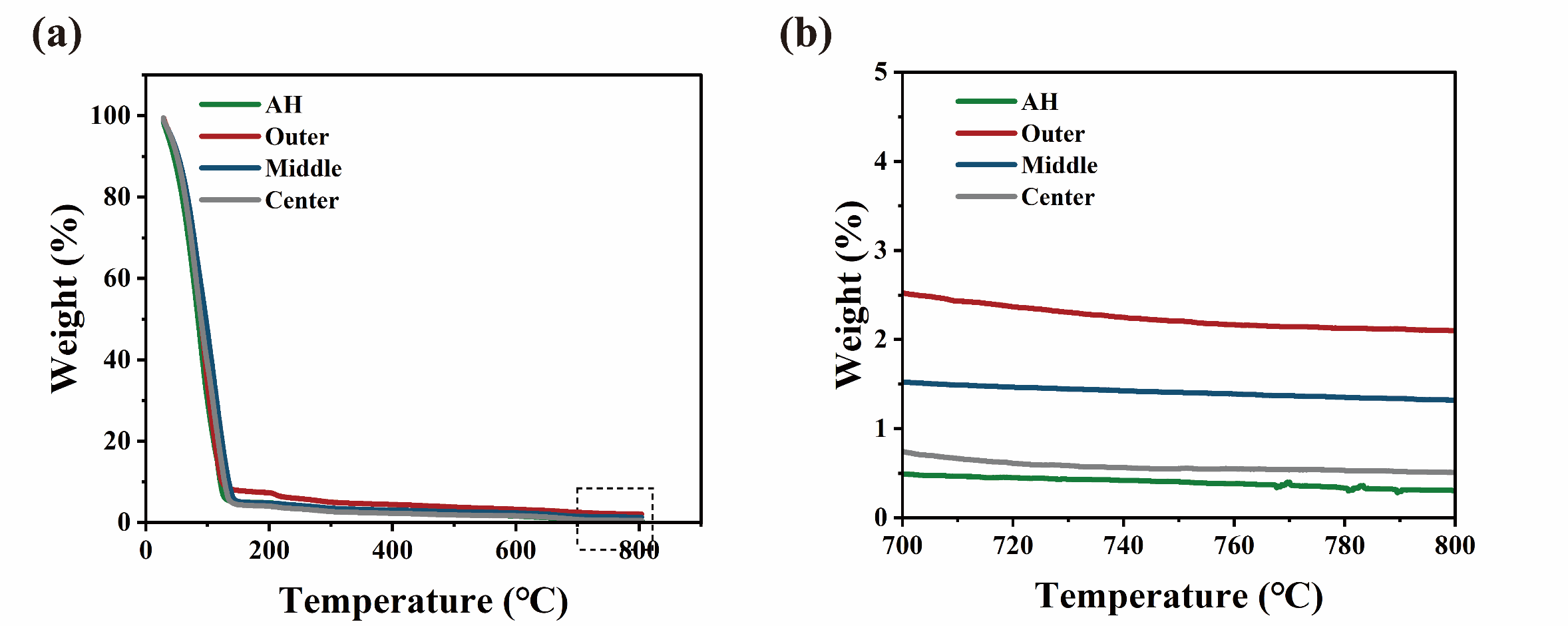 |
| --- |
| **Figure S6** TGA analysis of the wet LPH. (a) TGA analysis curve of the wet LPH (sample I). (b) Magnified of TGA analysis curve in (a). |

| 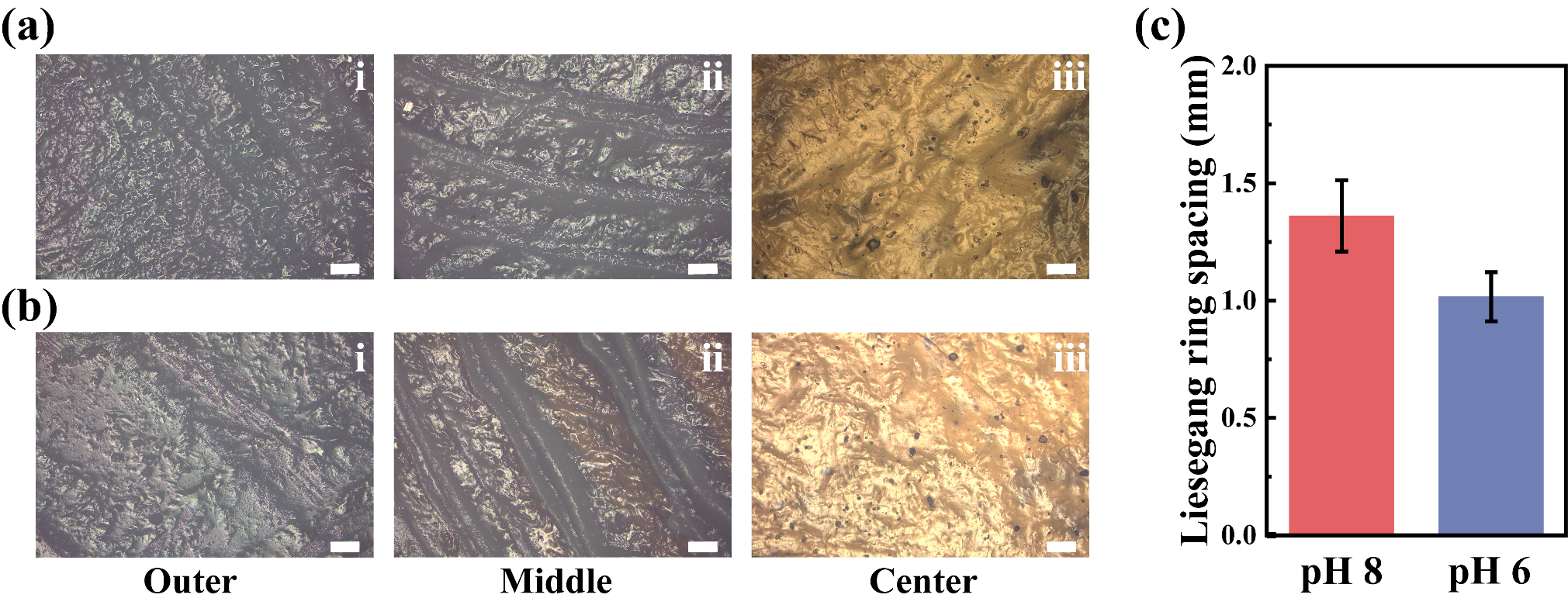 |
| --- |
| **Figure S7** Polarizing microscope images of the LPH and Liesegang ring spacing analysis. (a, b) Polarizing microscope images of the LPH at pH 6 (sample III) and pH 8 (sample I) were obtained for comparative analysis. Scale bar: 200 μm. (c) Comparative analysis of Liesegang ring spacing between the sample I and the sample III. |

| 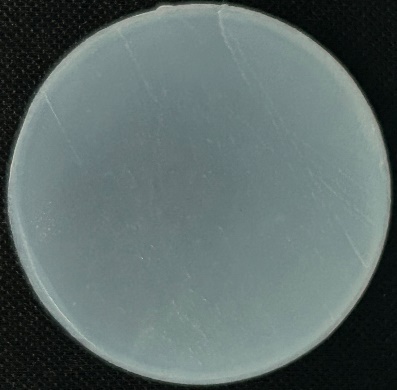 |
| --- |
| **Figure S8** Mineralized hydrogel complex photograph at pH 7.3. |

| 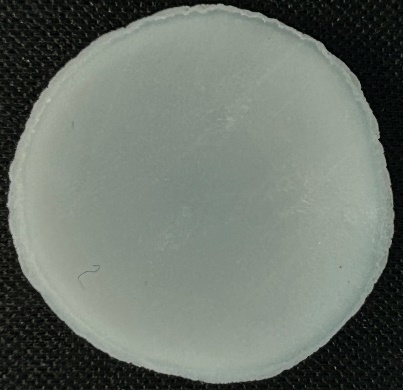 |
| --- |
| **Figure S9** Mineralized hydrogel complex photograph at 4 °C. |

| 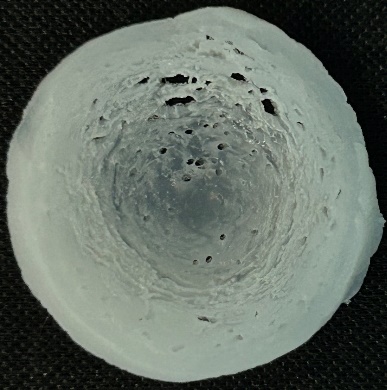 |
| --- |
| **Figure S10** Mineralized hydrogel complex photograph under the condition of 1% alginate. |

| 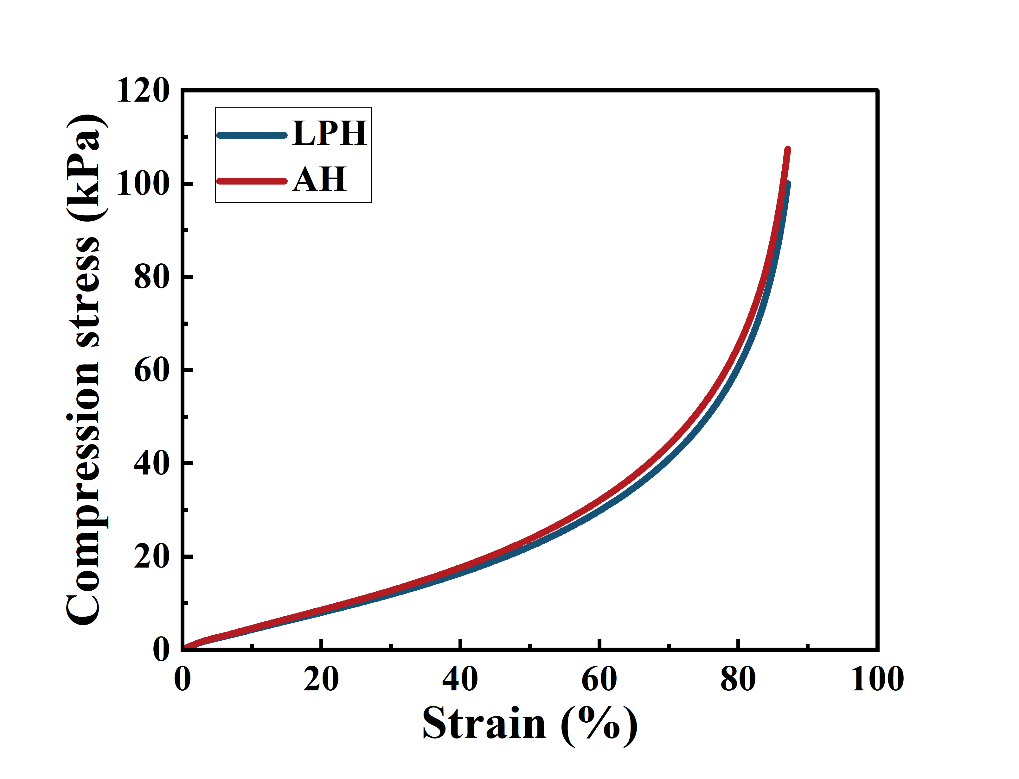 |
| --- |
| **Figure S11** Comparative analysis of compression stress between the AH and the LPH. |

**Table S1** Fabrication formulation of mineralized hydrogels.

|  | alginate (%) | CaCl_2_·2H_2_O | Na_2_HPO₄ | pH | temperature | LPH |
| --- | --- | --- | --- | --- | --- | --- |
| 1 | 2 | 0.1 M | 0.1 M | 8 | RT | No |
| 2 | 2 | 0.25 M | 0.1 M | 8 | RT | No |
| 3 | 2 | 0.5 M | 0.1 M | 8 | RT | No |
| 4 | 2 | 1 M | 0.1 M | 8 | RT | Yes |
| 5 | 2 | 1 M | 0.1 M | 6 | RT | Yes |
| 6 | 2 | 1 M | 0.1 M | 9 | RT | No |
| 7 | 2 | 1 M | 0.01 M | 8 | RT | No |
| 8 | 2 | 1 M | 0.25 M | 8 | RT | Yes |
| 9 | 2 | 1 M | 0.1 M | 8 | 4°C | No |
| 10 | 1 | 1 M | 0.1 M | 8 | RT | No |

Annotation: Room temperature is abbreviated RT, Liesegang pattern is abbreviated LP. The formulation 4 is a typical sample (sample I). The formulation 5 represents sample III. The formulation 8 represents sample II.

**Table S2** Primers sequence of osteogenesis relative genes.

| Gene | Forward primer (5'-3') | Reverse primer (5'-3') |
| --- | --- | --- |
| GAPDH | GGCACAGTCAAGGCTGAGAATG | ATGGTGGTGAAGACGCCAGTA |
| ALP | ACAATGAGATGCGCCCAGAG | CATGTACTTCCGGCCACCAC |
| RUNX2 | TTCGTCAGCGTCCTATCAGTTC | CTTCCATCAGCGTCAACACC |
| Col1α | GTACATCAGCCCAAACCCCA | TCGCTTCCATACTCGAACTGG |
| OCN | GCAGACCTAGCAGACACCAT | TTGGACATGAAGGCTTTGTCA |
| TGF-*β*1 | CAGAACCCCCATTGCTGTCC | AAGACAGCCACTCAGGCGTA |
